# Social Media Addiction, Perceived Stress, Emotional Intelligence, and Cyberbullying Among Thai Adolescents During the Transition from the COVID-19 Pandemic to the Endemic Phase

**DOI:** 10.3390/ijerph23040528

**Published:** 2026-04-18

**Authors:** Sasicha Rodpet, Tusana Thaweekoon, Wilai Napa

**Affiliations:** 1 Master of Nursing Science Program in Psychiatric and Mental Health Nursing, Ramathibodi School of Nursing, Faculty of Medicine Ramathibodi Hospital, Mahidol University, Bangkok 10400, Thailand; sasicha.rod@student.mahidol.ac.th; 2 Ramathibodi School of Nursing, Faculty of Medicine Ramathibodi Hospital, Mahidol University, Bangkok 10400, Thailand; wilai.nap@mahidol.ac.th

**Keywords:** cyberbullying, social media addiction, perceived stress, emotional intelligence, adolescents, COVID-19, endemic transition, Thai youth

## Abstract

**Highlights:**

**Public health relevance—How does this work relate to a public health issue?**

- Cyberbullying is a recognized public health concern among adolescents globally; the COVID-19 pandemic substantially intensified digital engagement and associated online risks, yet whether these effects have persisted into the endemic phase remains insufficiently examined in Southeast Asian contexts.
- Among 416 Thai secondary school students surveyed during the pandemic-to-endemic transition (2023), 66.4% reported some form of cyberbullying involvement, and 32.2% were classified as bully-victims, indicating that pandemic-induced digital behavioral changes have endured well beyond the acute crisis period.

**Public health significance—Why is this work of significance to public health?**

- Social media addiction and perceived stress were each significantly and positively correlated with both cyberbullying perpetration and victimization, while emotional intelligence demonstrated modest but statistically significant protective effects, suggesting distinct psychosocial patterns associated with different cyberbullying roles in a post-pandemic Southeast Asian adolescent population.
- Over one-third (34.4%) of Thai adolescents were classified as high risk for social media addiction, substantially exceeding pre-pandemic global estimates of 5–15%, indicating that pandemic-driven increases in digital dependency may be associated with sustained conditions conducive to ongoing cyberbullying involvement.

**Public health implications—What are the key implications or messages for practitioners, policymakers, and/or researchers in public health?**

- Practitioners and policymakers should prioritize multi-tiered, school-based interventions that concurrently address social media addiction, evidence-based stress management, and emotional intelligence development to reduce persistent cyberbullying risk in the post-pandemic adolescent population.
- Researchers should develop and evaluate culturally adapted prevention frameworks tailored to Southeast Asian contexts, where cyberbullying trends diverged markedly from Western patterns during and after the pandemic, and sustained cross-national surveillance is needed to track post-pandemic cyberbullying trajectories.

**Abstract:**

The COVID-19 pandemic significantly increased adolescent digital engagement, but whether the rise in cyberbullying persists beyond the crisis is not well understood, especially in Southeast Asia. This study examined social media addiction, perceived stress, emotional intelligence, and cyberbullying among 416 Thai secondary students (grades 7–12) during the pandemic-to-endemic transition (June–October 2023). Participants completed validated Thai-language instruments assessing cyberbullying, social media addiction, perceived stress, and emotional intelligence. Results showed 66.4% of adolescents were involved in cyberbullying, with 32.2% as bully-victims. Social media addiction correlated with cyberbullying perpetration (r_s_ = 0.33, *p* < 0.001) and victimization (r_s_ = 0.22, *p* < 0.001), as did perceived stress (r_s_ = 0.20 and 0.29; *p* < 0.001). Emotional intelligence showed negative correlations with cyberbullying perpetration (r_s_ = −0.15, *p* = 0.002) and victimization (r_s_ = −0.10, *p* = 0.048). Over one-third (34.4%) were at high risk for social media addiction. These findings indicate that during the pandemic-to-endemic transition, Thai adolescents showed elevated cyberbullying involvement, high social media addiction, and moderate-to-high stress—a profile consistent with sustained digital risk. These results highlight the need for integrated interventions that address digital wellness, stress management, and the development of emotional intelligence among Thai adolescents.

## 1. Introduction

The advent of the technological era has transformed society into a digital ecosystem, significantly altering adolescents’ communication patterns. Globally, adolescents are the heaviest internet users, with 95% of those aged 13–17 in developed countries owning smartphones [1], and those in middle-income countries spend 6–8 h daily on online activities [2]. The COVID-19 pandemic substantially accelerated this trajectory: average daily digital media consumption rose from 7.7 to 10.5 h [3], and the World Health Organization has identified excessive screen time as a public health concern, with problematic social media use affecting an estimated 5–15% of the global population [4]. These conditions have collectively intensified the risk of cyberbullying.

Cyberbullying has emerged as a significant public health concern alongside the expansion of digital communication technologies. It is characterized by intentional and repeated harm inflicted through electronic devices, often involving a power imbalance between perpetrator and victim [5], and encompasses threatening messages, dissemination of harmful content, online rumors, impersonation, and exclusion from online communities [6]. Unlike traditional bullying, cyberbullying is distinguished by perpetrator anonymity, permanence of content, potential to reach an unlimited audience, and reduced awareness of victim distress [7]. Researchers delineate two principal roles: cyberbullying perpetration, involving the deliberate infliction of harm through digital platforms [8], and cybervictimization, referring to being targeted online and experiencing psychological distress, anxiety, depression, and impaired social functioning [6].

### 1.1. Prevalence of Cyberbullying and the Pandemic-to-Endemic Research Gap

Global cyberbullying prevalence has varied substantially across contexts and time periods. Pre-pandemic estimates ranged from 6% to 46.3% for cyberbullying victimization and from 2.9% to 21.7% for perpetration [9,10,11,12,13]. During the pandemic (2020–2022), rates increased in many Asian countries and Australia, attributable to heightened online activity during lockdowns, whereas Western nations—including the United States, Canada, and parts of Europe—showed stable or slightly decreased rates despite increased screen time [9,10,14,15,16]. A pooled global meta-analysis reported pandemic-era prevalence of 16% for overall cyberbullying, 18% for cyberbullying victimization, and 11% for perpetration [12]. Emerging post-pandemic data from China and Greece indicate persistently elevated or increasing rates, with cyberbullying victimization reaching 17.7% and 11.6%, respectively [13,17].

In Thailand specifically, cyberbullying increased markedly during the pandemic. A 2021 national school-based survey reported 17% of male and 13% of female students experienced cyberbullying in the preceding year [18], while other studies employing different methodologies reported substantially higher rates, reaching 70.7% during the acute pandemic phase [19]. A systematic review of Asian countries identified Thailand as having one of the highest reported cyberbullying victimization rates at 49.2% [9]. Notably, however, existing Thai research has concentrated predominantly on the acute pandemic phase, leaving the post-pandemic endemic transition—a period during which adolescents are simultaneously re-adjusting psychologically, socially, and academically—largely unexamined. Whether the pandemic-induced surge in cyberbullying has persisted, stabilized, or declined as Thailand transitions to endemic status remains an important and unresolved empirical question.

In reviewing the scholarly literature on factors associated with cyberbullying perpetration and victimization among adolescents, three psychosocial variables emerge as particularly salient: social media addiction, perceived stress, and emotional intelligence. These factors have been examined across pre-, during-, and post-pandemic periods, and their relationships with cyberbullying are detailed below.

### 1.2. Social Media Addiction and Cyberbullying

Social media addiction is a behavioral addiction characterized by excessive usage, which subsequently impairs daily functioning. It adheres to the components model of addiction, sharing characteristics with substance addictions, including salience, mood modification, tolerance, withdrawal symptoms, conflict, and relapse [20]. This form of addiction manifests when individuals are unable to regulate their usage, exhibit preoccupation, utilize social media to escape problems, experience anxiety in the absence of access, and progressively increase the duration of use [21]. Social media addiction is closely linked to an increase in both the perpetration and victimization of cyberbullying, especially during the COVID-19 pandemic, with some evidence suggesting that these risks persist in the post-pandemic period. Even before the pandemic, there was a rising trend in cyberbullying, with social media usage identified as a primary risk factor [10,22]. During the pandemic, both social media addiction and cyberbullying incidents notably increased, with some studies reporting higher perpetration rates and either unchanged or slightly reduced victimization rates, possibly due to shifts in social dynamics and online behaviors [10,22,23,24,25,26]. In the post-pandemic context, while certain risk factors remain, the intensity of the relationship between social media addiction and cyberbullying may decrease as in-person interactions resume; however, digital risks continue to be elevated [27,28].

### 1.3. Perceived Stress and Cyberbullying

Perceived stress refers to how individuals appraise situations in their lives as being stressful. It encompasses the extent to which life circumstances are perceived as unpredictable, uncontrollable, and overwhelming. Rather than measuring objective stressful events, perceived stress captures the subjective evaluation of stress experienced, reflecting whether environmental demands exceed one’s perceived ability to cope [29]. Perceived stress is closely linked to both the perpetration and victimization of cyberbullying among adolescents. These connections became more pronounced during the COVID-19 pandemic and have remained significant in the post-pandemic era. Even before the pandemic, cyberbullying rates were on the rise, with stress and poor mental health contributing to increased victimization and perpetration [10,14]. During the pandemic, several studies noted a surge in cyberbullying perpetration and victimization, attributed to heightened stress, anxiety, and increased online activity [26,30,31]. However, meta-analyses suggest that overall cyberbullying rates remained stable or even slightly decreased, although stress continued to be a significant risk factor [12,14]. In the post-pandemic context, elevated stress and its association with cyberbullying persist, with recent data showing a high prevalence and strong links to mental health issues such as anxiety, depression, and PTSD [13,17].

### 1.4. Emotional Intelligence and Cyberbullying

Emotional intelligence was first formally conceptualized by Salovey and Mayer [32] as a set of interrelated abilities encompassing the accurate perception and appraisal of emotion in oneself and others, the facilitative use of emotional information to enhance adaptive thinking, the understanding of how emotions develop and interact over time, and the reflective regulation of emotion to promote intellectual and personal growth. Building on this foundational model, Goleman [33] extended the construct to include applied competencies that facilitate effective interpersonal functioning and adaptive behavior across social contexts. Emotional intelligence is consistently linked to reduced cyberbullying perpetration and victimization among adolescents, with this protective effect remaining significant before, during, and after the COVID-19 pandemic. Lower levels of emotional intelligence, particularly in emotional regulation and clarity, are associated with a higher likelihood of involvement in cyberbullying, either as a victim, perpetrator, or both [34,35,36,37,38,39,40]. Adolescents with higher emotional intelligence tend to experience fewer adverse psychological outcomes when victimized and are less likely to engage in aggressive online behaviors [37,38]. The COVID-19 pandemic, with its increased reliance on digital communication, has intensified the prevalence and impact of cyberbullying, making the role of emotional intelligence even more crucial during and after this period [41,42].

The present study is theoretically grounded in a risk and protective factor framework [43], in which social media addiction and perceived stress function as psychosocial risk factors that heighten adolescents’ involvement in cyberbullying, while emotional intelligence serves as a protective factor that may attenuate these risks. Within this framework, risk factors increase the probability of adverse outcomes, whereas protective factors buffer or moderate the impact of risk exposure on adjustment [44]. Critically, these three constructs are not independent of one another in their operation. Social media addiction intensifies adolescents’ digital immersion, thereby expanding both the frequency and duration of exposure to online environments in which cyberbullying perpetration and victimization occur [20,21]. Prolonged and compulsive platform engagement, in turn, amplifies stress through social comparison, harassment exposure, and disrupted sleep and self-regulation [26,45]. Perceived stress, once elevated, further compromises the emotional regulatory capacities that underpin emotional intelligence, rendering adolescents less able to inhibit impulsive online aggression or recognize and protect themselves from victimization [34,46]. Conversely, emotional intelligence may buffer the translation of stress and addictive engagement into cyberbullying involvement by facilitating adaptive coping, empathic restraint, and effective help-seeking behavior [37,39]. These interrelationships suggest that examining each variable in isolation—as much prior research have done—may underestimate the complexity of the psychosocial pathways through which cyberbullying risk accumulates. Importantly, the pandemic-to-endemic transition represents a period during which all three constructs may have been simultaneously elevated or destabilized: social media dependency intensified during lockdowns [3,4], academic and social stressors persisted into the endemic phase [13,17], and the disruption of school-based socialization may have curtailed the development and practice of emotional competencies [41,47]. Examining these variables in concert, in relation to both cyberbullying perpetration and victimization, is therefore essential to characterize the composite psychosocial risk profile of Thai adolescents during this transition period.

This study, therefore, aims to investigate the relationships between social media addiction, perceived stress, and emotional intelligence with cyberbullying perpetration and victimization among adolescents during the transition from the COVID-19 pandemic to the endemic phase, with a view to elucidating the distinct psychosocial pathways underlying each cyberbullying role. This study tested the following hypotheses:

**H1–H2:**  *Social media addiction would be significantly and positively associated with cyberbullying perpetration (H1) and victimization (H2).*

**H3–H4:**  *Perceived stress would be significantly and positively associated with cyberbullying perpetration (H3) and victimization (H4).*

**H5–H6:**  *Emotional intelligence would be significantly and negatively associated with cyberbullying perpetration (H5) and victimization (H6).*

## 2. Materials and Methods

### 2.1. Population

The target population comprised secondary school students in Grades 7–12 (Mathayom 1–6) residing in a province in the Central region of Thailand during the transition period from the COVID-19 pandemic to the endemic phase.

### 2.2. Sample Size Calculation

The sample size was determined using G*Power 3.1 (Heinrich Heine University Düsseldorf, Düsseldorf, Germany) [48], with the following parameters: Pearson’s correlation, a confidence level of 0.05, a power of 0.80, and two-tailed testing. Previous research has reported correlations between variables and game addiction ranging from 0.15 to 0.60. Using a conservative estimate of 0.15, the required sample size was 346. After accounting for incomplete responses by recruiting an additional 20% and excluding incomplete questionnaires.

### 2.3. Sampling Method

The present study employed a multistage sampling procedure, comprising the following steps:

(1) A comprehensive list of secondary schools under the jurisdiction of the Secondary Educational Service Area Office Region 3 was compiled. This administrative region encompasses two provinces.
(2) One provincial secondary educational service area was subsequently selected. Within this area, schools under the jurisdiction of the Secondary Educational Service Area Office were organized into three educational clusters, each comprising six schools, yielding a total of 18 schools.
(3) One school was then selected from each of Clusters 1 and 3, resulting in a total of two participating schools. School 1, drawn from Cluster 1, was located within the urban district, while School 2, drawn from Cluster 3, was situated in a district outside the urban area.
(4) The proportional sample size was subsequently calculated for each of the two schools. School 1 consisted of 11 classrooms per grade level for Grades 7–9 (Mathayom 1–3) and 10 classrooms per grade level for Grades 10–12 (Mathayom 4–6), with approximately 40 students per classroom. School 2 consisted of 15 classrooms per grade level for Grades 7–9 and 17 classrooms per grade level for Grades 10–12, with approximately 37 students per classroom. Based on a proportional allocation, the calculated sample sizes were 162 participants from School 1 and 255 participants from School 2, yielding a total sample of 416 participants.
(5) Once the overall sample size for each school was determined, the number of participants required from each grade level was calculated through proportional allocation.
(6) Classrooms were then selected by means of simple random sampling. In School 1, one classroom per grade level was randomly selected, whereas in School 2, two classrooms per grade level were selected at random.

### 2.4. Research Setting

The present study was conducted in two secondary schools in a province in Thailand’s Central region, operating under the Office of the Basic Education Commission (OBEC) as general education schools. School 1 was classified as a large school, enrolling 2162 students, and School 2 was classified as an extra-large school, enrolling 3602 students. Regarding information technology and student welfare policies, both schools permitted unrestricted use of mobile phones and internet access on school premises. Regarding mental health support services, both School 1 and School 2 provided guidance counselors and homeroom teachers who served as primary contacts for mental health consultation and referral, as well as for communicating mental health-related information to students’ parents or guardians. Notably, a proportion of teachers at School 2 received formal training in Cognitive Behavioral Therapy (CBT) delivered by a government agency.

### 2.5. Sampling Criteria

The following inclusion and exclusion criteria were applied.

Inclusion Criteria

(1) Aged between 11 and 19 years of any gender.
(2) Able to communicate, read, and write in the Thai language.
(3) Currently residing in a province in the Central region of Thailand.
(4) Willing to participate in the research study, with written consent obtained from a parent or legal Guardian authorized to provide parental permission.

Exclusion Criteria

Participants who chose to discontinue completion of the questionnaire or who voluntarily withdrew from the study at any point was excluded from the analysis.

### 2.6. Research Instruments

This study employed a self-administered questionnaire as the primary data collection instrument, comprising five sections.

Section 1: Demographic Questionnaire. This section was developed by the researchers to elicit participants’ general background information, including sex, age, educational level, cumulative grade point average (GPA), cohabiting individuals, family atmosphere, parental marital status, relationships with parents and peers, and COVID-19 infection history within the family. In addition, participants were presented with a predetermined checklist of 20 common stressors identified from the literature on Thai adolescent mental health (e.g., academic difficulty, unmet performance expectations, interpersonal conflicts, financial concerns); participants selected all applicable items. Social media behavior items similarly employed structured response formats with predefined categories (e.g., purpose of social media usage, social media platforms used, average daily time spent on social media).

Section 2: The Cyber-aggression Perpetration and Victimization Scale, developed by Shapka and Maghsoudi [49], was translated into Thai by Anuroj and Pityaratseathien [50]. This instrument consists of 18 items, divided into two categories: Cyberbullying Perpetration (9 items) and Cybervictimization (9 items). Responses are recorded on a 5-point Likert scale, ranging from never (0) to always (4), with summed total scores ranging from 0 to 36; higher summed scores indicate a greater frequency of cyberbullying perpetration or victimization. The scale demonstrated Cronbach’s alpha of 0.80 for perpetration and 0.84 for victimization. An item rated as ‘Sometimes’ or higher signifies cyberbullying perpetration or victimization. Data were categorized into four groups: (1) cyberbullying perpetration, (2) cyberbullying victimization, (3) both perpetration and victimization, and (4) those not involved in cyberbullying.

Section 3: The Social-Media Addiction Screening Scale, developed by Chanpen et al. [51], is a self-administered tool comprising 16 items that assesses the three main components of behavioral addiction: prioritization, impaired control, and negative consequences. This scale uses a 4-point Likert scale, ranging from 0 (“not at all”) to 3 (“completely”). The total score ranges from 0 to 48, with higher scores indicating a greater level of social media addiction. Scores are classified into low risk (0–15), moderate risk (16–30), and high risk (31–48). Reliability analysis produced Cronbach’s alpha coefficient of 0.88.

Section 4: The Perceived Stress Scale, initially developed by Cohen et al. [29], was translated into Thai by Wongpakaran and Wongpakaran [52] to assess stress levels over the past month. This tool consists of 10 items, each rated on a 5-point Likert scale, ranging from never (0) to very often (4). The total score ranges from 0 to 40, with higher scores indicating greater stress levels. Scores are classified as low (0–13), moderate (14–26), or high (27–40). In this study, Cronbach’s alpha was 0.82.

Section 5: The Emotional Intelligence Assessment scale, developed by the Department of Mental Health, Ministry of Public Health [53], was selected for the present study based on four considerations. First, the instrument is theoretically grounded in Goleman’s [33] multidimensional model of emotional intelligence, which delineates personal competence (self-awareness, self-regulation, and motivation) and social competence (empathy and social skills) as the core constituents of adaptive emotional functioning—a framework directly relevant to adolescent cyberbullying dynamics. Second, the Thai adaptation operationalizes Goleman’s constructs through three culturally contextualized domains—Good (emotional regulation, empathy, and social responsibility), Smart (self-awareness, motivation, decision-making, and problem-solving), and Happy (capacity for fulfilling life engagement)—thereby preserving theoretical fidelity while reflecting the sociocultural context of the target population. Third, the instrument has been previously validated for use with Thai adolescents aged 12–60 years, ensuring its construct validity for the current sample [53]. Fourth, the scale demonstrates robust internal consistency (overall Cronbach’s α = 0.88), with acceptable domain-level reliability (Good: α = 0.74; Smart: α = 0.72; Happy: α = 0.79), supporting its suitability for correlational analyses. The instrument comprises 52 items rated on a four-point Likert-type scale, ranging from 1 (not true at all) to 4 (very true of me), yielding scores from 52 to 208; higher scores denote greater emotional intelligence. Classifications are below average (<140), average (140–170), and above average (>170).

### 2.7. Data Collection

Data were collected from two secondary schools situated in a province in central Thailand between June and October 2023, a period marked by Thailand’s transition from a pandemic to an endemic phase. The procedures were as follows: (1) Upon receiving permission from the school principal, the researcher coordinated with relevant teachers, provided a detailed explanation of the research, and scheduled the date and time for data collection. (2) On the scheduled date, the researcher met with the sample to introduce themselves, elucidate the research objectives and participants’ rights, and distribute parental consent forms for students under 18 years of age. (3) On the day of data collection, the researcher collected the parental consent forms, obtained participants’ signatures on assent forms, and explained the procedure for completing the questionnaire, which took approximately 30 min. Participants subsequently placed the completed questionnaire in a designated collection box.

### 2.8. Ethical Consideration

The Ethical Review Committee of Ramathibodi Hospital, Faculty of Medicine, Mahidol University, approved this study (approval code: COA MURA2023/121). Written informed consent was obtained from both students and parents prior to their involvement. Participation was entirely voluntary and without financial compensation, and participants retained the right to withdraw at any point without facing academic penalties. Data confidentiality was maintained through the use of coded identifiers. Participants who experienced distress were directed to school counselors. To minimize response bias, participants were given the option to submit their questionnaires anonymously.

### 2.9. Data Analysis

A total of 193 questionnaires were collected from School 1, of which 19 were deemed incomplete and subsequently excluded. From School 2, 297 questionnaires were collected, of which 55 were incomplete and excluded from analysis. The remaining complete and usable questionnaires totaled 416, and these were carried forward for statistical analysis. Data analysis was conducted using IBM SPSS Statistics version 29 (IBM Corp., Armonk, NY, USA), with the following statistical techniques: (1) Descriptive statistics (percentages, ranges, means, standard deviations) were used to assess participant demographics and study variables; (2) Variable distribution was evaluated using the Kolmogorov–Smirnov test, which showed that social media addiction, perceived stress, emotional intelligence, cyberbullying perpetration, and cyberbullying victimization were not normally distributed (Z = 0.052, 0.069, 0.057, 0.189, 0.139; *p* = 0.009, 0.000, 0.003, 0.000, and 0.000, respectively). This finding justifies the use of non-parametric statistical methods. Consequently, Spearman’s rank-order correlation coefficient was used to explore relationships among the variables.

Demographic characteristics are summarized in Table 1. Females constituted a slight majority. The age distribution was concentrated in the 14–16-year-old bracket, and students were distributed roughly equally across the lower and upper secondary levels. Most participants resided in nuclear family arrangements, identified as Buddhist, and reported academic performance in the upper GPA range. Family-related COVID-19 illness was common, whereas mortality due to COVID-19 was rare.

Table 2 presents the stress sources endorsed by participants. Academic stressors predominated, with coursework difficulty and unmet performance expectations being the most frequently cited concerns, followed by insufficient study time. Interpersonal stressors, including competitive social pressure and appearance dissatisfaction, formed a secondary cluster, whereas family-related stressors, such as household conflict and financial hardship, were comparatively infrequent. This school-centered stress profile contextualizes the moderate-to-high perceived stress levels observed across most participants in the subsequent analyses.

Table 3 summarizes digital engagement characteristics across the sample. Daily internet use was nearly universal, with heavy-frequency access predominating. Notably, a substantial proportion of participants spent more than four hours daily on social media —a pattern directly relevant to addiction risk. TikTok and Facebook were the most prevalent platforms. Parental supervision was predominantly occasional or absent, a finding with implications for protective monitoring. Despite relatively high rates of self-reported privacy configuration, nearly half of the participants shared account credentials with acquaintances, indicating potential security vulnerability.

## 3. Results

### 3.1. Descriptive Statistics of Main Variables

Table 4 presents descriptive statistics for all primary study variables. Cyberbullying victimization scores were notably higher in both mean and variability relative to perpetration scores, suggesting that being targeted was more prevalent and more heterogeneous across the sample than perpetrating. Social media addiction scores spanned the full possible range, reflecting substantial individual variation. Perceived stress clustered in the moderate range, while emotional intelligence scores indicated that most participants fell within the average band of competence.

Table 4 details cyberbullying involvement across the four classificatory categories. Overall, 66.4% of participants reported some form of cyberbullying involvement—a rate substantially exceeding both pre-pandemic global estimates and recent post-pandemic figures from Western contexts. The largest single group (32.2%) comprised bully-victims, indicating that dual-role involvement was more common than either pure perpetration or pure victimization. Only one-third of the sample reported no involvement whatsoever.

The risk profiles of the three psychosocial variables (Table 4) indicate that social media addiction was distributed across all three risk categories with notable concentration in the moderate-to-high range. Perceived stress was heavily concentrated at the moderate level, with relatively few participants reporting either low or high stress. The distribution of emotional intelligence was skewed toward the average band, with a minority of participants achieving above-average scores—a pattern that limits the availability of this protective resource across the sample.

### 3.2. Correlation Analysis

Spearman’s rank-order correlations between the psychosocial variables and cyberbullying outcomes are presented in Table 5. All associations were statistically significant. Social media addiction demonstrated the strongest link with cyberbullying perpetration (r_s_ = 0.33) relative to its association with victimization, whereas perceived stress showed the reverse pattern—a more pronounced correlation with cyberbullying victimization (r_s_ = 0.29) than with perpetration. These divergent patterns suggest that the two cyberbullying roles may be driven by partially distinct psychosocial mechanisms. Emotional intelligence exhibited modest but significant negative correlations with both outcomes, consistent with a protective function, though the magnitude of these associations was weaker than those observed for the risk variables.

The observed correlation patterns support the hypothesized relationships, indicating that social media addiction and perceived stress serve as risk factors for involvement in cyberbullying, while emotional intelligence seems to act as a protective factor. The varying strength of associations between the variables and the roles of perpetration versus victimization suggest that distinct underlying mechanisms may influence these different forms of cyberbullying involvement. Notably, social media addiction showed a stronger correlation with perpetration, whereas perceived stress demonstrated a more pronounced association with victimization, suggesting potentially divergent pathways to various forms of cyberbullying engagement.

## 4. Discussion

### 4.1. Temporal Trends in Cyberbullying Prevalence

The prevalence patterns observed in our study reflect a complex interplay of factors that have evolved throughout the pandemic timeline. Prior to the COVID-19 pandemic, global cyberbullying victimization rates ranged widely, from 6% to 46.3%, with perpetration rates between 2.9% and 21.7% [10,11,12]. These pre-pandemic baselines provide essential context for understanding the dramatic shifts that occurred during and after the pandemic period.

During the pandemic phase, our findings corroborate the substantial increase in cyberbullying prevalence documented across Asian countries. The systematic review by Sorrentino et al. [9] identified Thailand as having one of the highest reported cyberbullying victimization rates at 49.2%, consistent with Thongnopakun et al. [19], who reported a striking 70.7% prevalence during the pandemic period. This increase has been associated with the unprecedented rise in digital media consumption, which rose from 7.7 to 10.5 h daily [3], creating expanded opportunities for online harassment and victimization.

Interestingly, while Asian countries experienced increased cyberbullying rates during lockdowns, Western nations showed divergent patterns. Studies from the United States, Canada, and Europe reported stable or slightly decreased rates of cyberbullying despite increased screen time [10,14,15,16]. This geographical variation suggests that cultural factors, digital literacy levels, and pandemic response strategies may be associated with differences in the relationship between increased online activity and cyberbullying behaviors.

### 4.2. Post-Pandemic Persistence and the “New Normal”

Our study’s timing during the endemic transition phase in 2023 reveals concerning patterns of sustained high cyberbullying involvement. The persistence of elevated rates aligns with recent findings from China, where Wang et al. [13] reported 17.7% cyberbullying victimization and 8.1% perpetration rates following the restrictions, and Greece, where victimization rates increased from 4.0% in the pre-pandemic period to 11.6% in 2023 [17]. The elevated rates observed in our sample during the pandemic-to-endemic transition are consistent with post-pandemic surveillance data from other countries, suggesting that cyberbullying risk has not diminished as acute pandemic conditions have resolved. Whether these patterns reflect a durable structural shift in adolescent digital behavior or a transitional phenomenon requires longitudinal investigation.

### 4.3. Social Media Addiction as a Key Correlate of Cyberbullying

The strong correlation between social media addiction and both cyberbullying perpetration (r_s_ = 0.33, *p* < 0.001) and victimization (r_s_ = 0.22, *p* < 0.001) identified in our study (Table 5) aligns with the theoretical framework proposed by Kuss and Griffiths [21], which supports empirical findings observed during the pandemic. These associations mirror those reported by Topan et al. [45], who found significant positive correlations between technology addiction scores and both cyberbullying victimization and cyberbullying perpetration among Turkish adolescents. Similarly, Kucuk et al. [54] demonstrated that social media addiction was associated with an elevated risk of cyberbullying perpetration and cyberbullying victimization among Turkish adolescents.

The significant rise in screen time during the COVID-19 pandemic may be associated with the normalization of excessive social media use. The finding that 34.4% of participants were classified as high risk for social media addiction substantially exceeds pre-pandemic global estimates of 5–15% for problematic social media use [4]. This difference is consistent with reports of increased digital media consumption during the pandemic [3], although the present cross-sectional design does not permit attributing the observed addiction rates to pandemic-related behavioral changes within this sample. This prevalence aligns with Kucuk et al.’s finding [54] that 23.3% of adolescents were identified as social media addicts, with those using social media for more than three hours daily facing a 3.45 times higher risk of experiencing cyberbullying.

The significant positive associations between social media addiction and both cyberbullying perpetration (r_s_ = 0.33) and victimization (r_s_ = 0.22) are consistent with theoretical accounts proposing reciprocal relationships between problematic platform use and online aggression, as documented in prior longitudinal research [45,55]. However, the directionality of these associations cannot be established from the present cross-sectional data. The specific mechanisms underlying these associations—such as increased exposure to online conflict or reinforcement of platform engagement following negative experiences—warrant examination in prospective study designs. This pattern is particularly concerning in light of the high bully-victim prevalence observed in the present sample (32.2%; Table 4). These dual-role participants represent a particularly vulnerable population, as research indicates they are associated with compounded psychological distress compared to individuals who are solely perpetrators or victims [13]. The sustained post-pandemic elevations documented in Section 4.1 suggest that these pandemic-era digital habits have established durable behavioral patterns, reinforcing the need to examine additional psychosocial factors that may compound cyberbullying risk.

### 4.4. Perceived Stress as a Differential Correlate of Cyberbullying Perpetration and Victimization

The study found significant positive correlations between perceived stress and both cyberbullying perpetration (r_s_ = 0.20, *p* < 0.001) and cyberbullying victimization (r_s_ = 0.29, *p* < 0.001) among adolescents. The stronger correlation with victimization suggests different vulnerability mechanisms. Notably, 79.3% of participants reported experiencing moderate stress levels, with academic challenges being the primary source, as 68.0% indicated they faced excessive difficulty. These findings align with pandemic research. González-Cabrera et al. [46] noted baseline links between stress and cyberbullying before the pandemic using cortisol measurements. Geng and Lei [56] identified COVID-19-related stressors—like academic disruption and social isolation—associated with online aggression, influenced by fatalism and reduced self-compassion. Post-pandemic, Chu et al. [57] found ongoing stress–cyberbullying connections through expressive suppression and online disinhibition.

The relationship between stress and cyberbullying perpetration (r_s_ = 0.20) is consistent with frustration–aggression frameworks, wherein adolescents perceive situations as threatening or beyond their control, they may be more likely to engage in online aggression as a way to release their emotions [46]. Jiang et al. [30] demonstrated that pandemic-related stress, moderated by perceived safety and family cohesion, predicted cyberbullying perpetration among Chinese adolescents facing academic pressures similar to those experienced in our sample. The stronger association between perceived stress and cyberbullying victimization (r_s_ = 0.29) relative to perpetration (r_s_ = 0.20) suggests that stress may be differentially associated with vulnerability to online targeting, a pattern noted in prior research [34,58]. The behavioral and emotional processes through which stress may elevate victimization risks, such as emotional dysregulation in online interactions, were not directly assessed in this study and warrant investigation in future research. Ramadan et al. [58] reported that stress has been associated with reduced self-protective behaviors and the ability to seek support among high school students, with stressed adolescents being 2.3 times more likely to experience cybervictimization. Moreover, stress-associated inappropriate online expressions—such as emotional volatility and displays of vulnerability—have been observed to coincide with greater perpetrator targeting [34].

### 4.5. Protective Role of Emotional Intelligence

The study found significant negative correlations between emotional intelligence and both cyberbullying perpetration (r_s_ = −0.15, *p* = 0.002) and cyberbullying victimization (r_s_ = −0.10, *p* = 0.048) among Thai adolescents. This suggests that higher emotional intelligence may be associated with lower involvement in cyberbullying, suggesting that distinct patterns may be associated with different cyberbullying roles. Notably, 56.3% of participants demonstrated average emotional intelligence, while only 13.9% exhibited above-average emotional intelligence. These findings align with international evidence spanning the pre-pandemic, pandemic, and post-pandemic periods. Martínez-Monteagudo et al. [35] identified that deficiencies in emotional regulation and clarity predict involvement in cyberbullying. During the pandemic, Yudes et al. [37] demonstrated that emotional intelligence moderated the relationship between problematic internet use and cyberbullying. These findings are consistent with prior research reporting that adolescents with higher emotional intelligence engage in meaningfully less cyberbullying perpetration, even in contexts of elevated online activity [37]. Post-pandemic research by Quintana-Orts [59] confirmed the lasting protective effects of empathy and emotional regulation.

The stronger correlation between emotional intelligence and cyberbullying perpetration (r_s_ = −0.15) suggests the presence of regulatory associations. According to Goleman’s framework [33], emotional self-awareness and regulation may be associated with reduced impulsive aggression during online conflicts. Moreta-Herrera et al. [39] demonstrated, using structural equation modeling, that emotion regulation accounted for 35% of the variance in cyberbullying perpetration. Adolescents with well-developed emotional intelligence exhibit greater empathy, which may be associated with a greater recognition of potential harm before engaging in aggressive behavior. In contrast, the relatively weak correlation between emotional intelligence and cyberbullying victimization (r_s_ = −0.10, *p* = 0.048) suggests a limited capacity for external protection. The relatively modest correlation between emotional intelligence and cyberbullying victimization (r_s_ = −0.10, *p* = 0.048) suggests that emotional intelligence may have a more limited association with cyberbullying victimization than with perpetration, consistent with prior observations that emotional competencies may be more strongly implicated in the regulation of aggressive behavior than in protection from external targeting [60]. However, whether emotional intelligence prevents initial cyberbullying victimization, mitigates its psychological consequences, or both cannot be determined from the present cross-sectional data. Villegas-Lirola [40] demonstrated, through a multilevel analysis, that emotional intelligence primarily protects against the psychological consequences of victimization rather than preventing victimization itself. Nonetheless, adolescents with higher emotional intelligence display improved boundary-setting and support-seeking behaviors, which may be associated with a reduced likelihood of repeated victimization [39].

### 4.6. Implications for Intervention

These findings carry direct implications for school-based prevention programming. Given that social media addiction showed the strongest association with perpetration (r_s_ = 0.33) and perceived stress with victimization (r_s_ = 0.29), a risk-stratified, multi-tiered model is recommended. At the universal tier, all students should receive classroom-based digital wellness instruction and social–emotional learning (SEL), covering healthy use habits, impulse regulation, and empathy, to address the normalization of excessive use observed in this sample (57.2% accessing social media more than 12 times daily). At the selective tier, small-group cognitive–behavioral stress management programs—incorporating mindfulness approaches culturally aligned with Buddhist practice—should target students exhibiting academic stress and heightened victimization vulnerability. At the indicated tier, individualized cognitive–behavioral therapy addressing online disinhibition and frustration–aggression responses, combined with family-based digital supervision interventions, is warranted for the 32.2% classified as bully-victims. Effective implementation further requires sustained teacher and counselor capacity-building, structured parental engagement, and routine outcome monitoring using validated Thai-language instruments to track program impact over time.

### 4.7. Limitations and Methodological Considerations

Some methodological limitations warrant consideration when interpreting the present findings. First, the exclusive use of bivariate correlation analyses precluded estimating the independent contribution of each predictor variable to cyberbullying outcomes; future studies should employ multivariate regression frameworks to disentangle the unique and shared variance attributable to social media addiction, perceived stress, and emotional intelligence.

Second, restricting the sample to two secondary schools in a single province in Central Thailand substantially limits the generalizability of the findings. Thailand exhibits considerable regional heterogeneity in socioeconomic conditions, internet infrastructure, and cultural norms governing online behavior. Adolescents in rural or remote provinces may face markedly different conditions—including constrained internet access, lower smartphone penetration, and limited school-based counseling resources—that could meaningfully alter both the prevalence and psychosocial correlations of cyberbullying. Accordingly, the reported prevalence estimates, and correlation coefficients should not be regarded as nationally representative, and extrapolation to other Thai regions or comparable Southeast Asian contexts should be undertaken with caution. Nationally stratified, multi-site sampling designs are needed to generate findings of greater ecological validity.

Third, exclusive reliance on self-report instruments introduces measurement bias that may compromise the accuracy of key estimates. Cyberbullying perpetration is particularly susceptible to underreporting due to social stigma and anticipated disciplinary consequences, potentially attenuating observed perpetration–predictor correlations. Emotional intelligence scores may be inflated by aspirational self-presentation, whereas retrospective perceived stress measures may not fully capture fluctuations in stress exposure over time. Future studies incorporating multiple informant approaches and objective stress indicators would strengthen the validity of findings in this domain.

### 4.8. Future Directions and Recommendations

The present findings point to several priorities for future research and practice. First, longitudinal designs are needed to track cyberbullying trajectories as Thailand and comparable middle-income countries advance further into the endemic phase, clarifying whether currently elevated prevalence reflects a transient post-pandemic adjustment or a durable behavioral shift. Second, future studies should employ hierarchical multiple regression or structural equation modeling to establish the relative contributions of social media addiction, perceived stress, and emotional intelligence to cyberbullying outcomes, moving beyond the bivariate associations permitted by the present cross-sectional design. Third, dedicated research is warranted on the bully-victim subgroup—comprising 32.2% of the present sample—given the compounded psychosocial risks faced by adolescents who occupy both perpetrator and victim roles concurrently. Finally, the pronounced divergence in cyberbullying trends between Asian and Western contexts underscores the need for cross-cultural comparative studies and culturally adapted prevention frameworks tailored to the specific sociocultural and digital access conditions of Southeast Asian adolescent populations.

## 5. Conclusions

This cross-sectional study found that 66.4% of Thai adolescents reported cyberbullying involvement—with 32.2% classified as bully-victims—during the pandemic-to-endemic transition, indicating that pandemic-associated digital behavioral changes have persisted beyond the acute crisis. Social media addiction and perceived stress were each positively correlated with both cyberbullying perpetration and victimization, while emotional intelligence demonstrated modest but statistically significant protective effects against both. The divergent patterns—social media addiction more strongly associated with perpetration (r_s_ = 0.33 vs. 0.22) and perceived stress more strongly linked to victimization (r_s_ = 0.29 vs. 0.20)—suggest distinct psychosocial pathways underlying different cyberbullying roles. Comprehensive, school-based interventions simultaneously addressing digital wellness, evidence-based stress reduction, and emotional intelligence development are warranted, supported by sustained collaboration among researchers, educators, policymakers, and mental health professionals to address cyberbullying as an enduring public health concern in the post-pandemic digital landscape.

## Figures and Tables

**Table 1:** Demographic Information of Participants (*n* = 416).

Demographic Characteristics	Number of Respondents	Percentage
Gender		
Male	176	42.3
Female	240	57.7
Age		
11–13 years	127	30.5
14–16 years	212	51.0
17–19 years	77	18.5
Education Level		
Grade 7	87	20.9
Grade 8	67	16.1
Grade 9	73	17.5
Grade 10	69	16.6
Grade 11	59	14.2
Grade 12	61	14.7
Grade Point Average (GPA)		
Below 2.00	2	0.5
2.01–2.51	32	7.7
2.51–3.00	56	13.5
3.01–3.50	120	28.8
3.51–4.00	206	49.5
Living Arrangements		
Independent living	6	1.4
Co-residence with peers	5	1.2
Co-residence with both parents	293	70.4
Residence with father only	19	4.6
Residence with mother only	63	15.1
Co-residence with extended family members or siblings	18	4.3
Co-residence with a romantic partner	3	0.7
Co-residence with family and a romantic partner	9	2.2
Family atmosphere		
Cohesive and harmonious	256	61.5
Occasional interpersonal conflict	153	36.8
Frequent interpersonal conflict	7	1.7
Marital status of parents		
Married or cohabiting	301	72.4
Separated	49	11.8
Divorced	59	14.2
Bereavement of one parent	7	1.7
Relationship with parents		
Excellent	200	48.1
Good	133	32.0
Moderately good	52	12.5
Moderately poor	22	5.3
Poor	9	2.2
Relationship with friends		
Excellent	208	50.0
Good	175	42.1
Moderately good	25	6.0
Moderately poor	6	1.4
Poor	2	0.5
Family history of COVID-19 infection		
Yes	384	92.3
No	32	7.7
Family history of death from COVID-19		
Yes	12	2.9
No	404	97.1

**Table 2:** Sources of Stress among Participants (*n* = 416).

Sources of Stress	Number of Respondents	Percentage
Perceived difficulty of academic coursework	283	68.0
Academic performance does not meet expectations	258	62.0
Insufficient time for examination preparation	215	51.7
Perceived competitive pressure or risk of exploitation by others	159	38.2
Dissatisfaction with personal physical appearance	148	35.6
Disappointment in romantic relationships	100	24.0
Limited autonomy in personal decision-making	97	23.3
Perceived social exclusion or lack of peer acceptance	85	20.4
Personal health concerns	82	19.7
Interpersonal difficulties with classmates	80	19.2
Absence of romantic interest from peers	78	18.8
Insufficient access to academic or personal advisors	72	17.3
Intrafamilial conflict	67	16.1
Family financial difficulties	65	15.6
Health concerns among family members	52	12.5
Personal financial indebtedness	38	9.1
Parental separation	35	8.4
Financial dependence on others	19	4.6
Necessity of part-time employment for financial self-support	10	2.4
Unsuccessful scholarship applications	4	1.0

**Table 3:** Social Media Behaviors of Participants (*n* = 416).

Social Media Usage Characteristics	Number of Respondents	Percentage
Purpose of Internet Usage		
Streaming media content (television programs, video clips, and films)/Music streaming	394	94.7
Social media platform use	380	91.3
Online gaming	354	85.1
Online communication, including phone calls	349	83.9
and messaging	345	82.9
Online information retrieval	290	69.7
Online learning	282	67.8
Online shopping	239	57.5
Academic or professional content creation	224	53.8
Read articles/e-books	185	44.5
Send and receive emails	155	37.3
Online financial transactions		
Purpose of Social Media Usage		
Entertainment viewing (videos, films, and series)	365	87.7
Online gaming	339	81.5
Interpersonal communication	337	81.0
Following social connections, public figures, and influencers	316	76.0
Content sharing (messages, photographs, and videos)	293	70.4
Following news, current events, and entertainment content	272	65.4
Online shopping	266	63.9
Peer exchange of information and content	190	45.7
Formation of new social connections	172	41.3
Location-based check-in	115	27.6
Social media platforms used		
Line	405	97.4
YouTube	401	96.4
Instagram	373	89.7
Facebook	369	88.7
TikTok	363	87.3
Twitter	290	69.7
Snapchat	34	8.2
WhatsApp	23	5.5
Tinder	8	1.9
Frequency of social media usage		
More than 12 times per day	238	57.2
10–12 times per day	86	20.7
7–9 times per day	55	13.2
4–6 times per day	35	8.4
1–3 times per day	2	0.5
Time periods of social media usage		
08:01–12:00	266	63.9
12:01–16:00	300	72.1
16:01–20:00	328	78.8
20:01–00:00 (midnight)	271	65.1
00:01–04:00	62	14.9
04:01–08:00	68	16.3
Average daily time spent on social media		
More than 17 h per day	58	13.9
9–16 h per day	178	42.8
1–8 h per day	177	42.5
Less than 1 h per day	3	0.7
Devices used to access social media		
Mobile phone/Smartphone	409	98.3
Computer/Laptop	213	51.2
Tablet	205	49.3
Total number of friends on social media		
Less than 10 people	57	13.7
11–50 people	93	22.4
51–100 people	48	11.5
101–200 people	54	13.0
201–500 people	81	19.5
501 people or more	83	20.0
Personal information disclosure on social media	72	17.3
Do not share any personal information	333	80.0
Share some personal information	11	2.6
Share a lot of personal information		
Frequency of changing passwords for social media accounts		
Never change	80	19.2
Rarely/Occasionally	302	72.6
Change frequently	34	8.2
Privacy settings for social media accounts		
Privacy settings not configured	106	25.5
Privacy settings configured	310	74.5
Sharing user accounts with others		
Do not share accounts with others	176	42.3
Share user accounts only with acquaintances	200	48.1
Share the user account publicly	40	9.6
Parental control of social media usage		
Absence of parental supervision	181	43.5
Intermittent parental supervision	228	54.8
Strict parental supervision	7	1.7

**Table 4:** Descriptive Statistics for Main Study Variables (*n* = 416).

Variable	*n*	%	M	SD	Range
Continuous variables	—	—	—	—	—
Cyberbullying perpetration	—	—	3.11	3.04	0–34
Cyberbullying victimization	—	—	5.10	4.13	0–30
Social media addiction	—	—	25.06	9.35	0–48
Perceived stress	—	—	21.26	4.88	3–36
Emotional intelligence	—	—	150.36	17.11	107–203
Cyberbullying involvement	—	—	—	—	—
Not involved	140	33.6	—	—	—
Perpetration only	31	7.5	—	—	—
Victimization only	111	26.7	—	—	—
Both (bully-victim)	134	32.2	—	—	—
Social media addiction risk level	—	—	—	—	—
Low risk (0–15)	118	28.4	—	—	—
Moderate risk (16–30)	155	37.3	—	—	—
High risk (31–48)	143	34.4	—	—	—
Perceived stress level	—	—	—	—	—
Low (0–13)	27	6.5	—	—	—
Moderate (14–26)	330	79.3	—	—	—
High (27–40)	59	14.2	—	—	—
Emotional intelligence level	—	—	—	—	—
Below average (<140)	124	29.8	—	—	—
Average (140–170)	234	56.3	—	—	—
Above average (>170)	58	13.9	—	—	—

Note. M = Mean; SD = Standard deviation; — = not applicable.

**Table 5:** Correlation between Social Media Addiction, Perceived Stress, Emotional Intelligence, and Cyberbullying Perpetration and Victimization among Adolescents (*n* = 416).

Variables	Correlation Coefficient (r_s_)	*p*-Value
Social Media Addiction		
Cyberbullying perpetration	r_s_ = 0.33	<0.001
Cyberbullying victimization	r_s_ = 0.22	<0.001
Perceived Stress		
Cyberbullying perpetration	r_s_ = 0.20	<0.001
Cyberbullying victimization	r_s_ = 0.29	<0.001
Emotional Intelligence		
Cyberbullying perpetration	r_s_ = −0.15	0.002
Cyberbullying victimization	r_s_ = −0.10	0.048

## Data Availability

The data presented in this study are available on request from the corresponding author due to ethical reasons.
